# Eucalyptol Ameliorates Neuroendocrine Stress-Aggravated Rheumatoid Arthritis Through Anti-inflammatory, Antioxidant, and Chondroprotective Mechanisms Associated with NF-κB and MAPK Signaling Pathways

**DOI:** 10.7759/cureus.107679

**Published:** 2026-04-24

**Authors:** Lavkush Tiwari, Nitu Nigam, Urmila Dhakad, Suyog Sindhu, Puneet Kumar, Ch. V Rao, Shubha Shukla, Suresh Babu

**Affiliations:** 1 Centre for Advanced Research, King George’s Medical University, Lucknow, IND; 2 Department of Rheumatology, King George’s Medical University, Lucknow, IND; 3 Department of Pharmacology, King George’s Medical University, Lucknow, IND; 4 Pharmacology Division, CSIR-National Botanical Research Institute, Lucknow, IND; 5 Division of Neuroscience, CSIR-Central Drug Research Institute (CDRI), Lucknow, IND; 6 Department of Pathology, King George’s Medical University, Lucknow, IND

**Keywords:** autoimmune synovitis, neuroendocrine stress response, nf-kb signaling pathway, restraint stress, : rheumatoid arthritis

## Abstract

Background: Rheumatoid arthritis (RA) is a lingering autoimmune disorder typified by synovial inflammation, oxidative imbalance, and advanced articular damage. Psychological and physical stress are known to exacerbate disease progression by enhancing inflammatory signaling and disrupting redox homeostasis. Although methotrexate remains a first-line therapy, its long-term use is accompanied by adverse effects, necessitating safer adjunctive strategies. Eucalyptol (1,8-cineole), a naturally occurring monoterpene, has demonstrated anti-inflammatory and antioxidant potential; however, its role in stress-aggravated RA remains inadequately explored.

Objective: To evaluate the protective effects of eucalyptol in restraint stress-augmented Complete Freund’s Adjuvant (CFA)-induced arthritis and to investigate its underlying mechanisms, both as monotherapy and in combination with methotrexate.

Methodology: Experimental arthritis was induced in Wistar rats using CFA, followed by chronic restraint stress for 28 days. Animals were treated with eucalyptol (50, 100, or 200 mg/kg), methotrexate (1 mg/kg), or their combination. Disease progression was assessed using clinical scoring, functional parameters, biochemical assays, cytokine profiling, nuclear factor-kappa B (NF-κB) and mitogen-activated protein kinase (MAPK) pathway analysis, gene expression studies, and histopathological evaluation.

Results: Restraint stress significantly aggravated arthritic severity, resulting in increased oxidative and nitrosative stress, elevated pro-inflammatory cytokines, activation of NF-κB/MAPK signaling, and enhanced joint damage. Eucalyptol treatment produced significant dose-dependent improvements across these parameters. Notably, it reduced tumor necrosis factor-α (TNF-α) and interleukin-6 (IL-6) levels while restoring antioxidant defenses. Combination therapy demonstrated the most pronounced therapeutic effect, with near normalization of multiple disease indicators.

Conclusions: Eucalyptol attenuates stress-exacerbated RA through integrated anti-inflammatory and antioxidant mechanisms. Its enhanced efficacy in combination with methotrexate highlights its potential as a complementary therapeutic strategy in RA management. Serum NF-κB and MAPK levels were used as surrogate markers of pathway involvement.

## Introduction

Rheumatoid arthritis (RA) is a lingering autoimmune disorder that primarily affects synovial joints, leading to persistent inflammation, synovial hyperplasia, and progressive cartilage and bone destruction [1-3]. Its pathogenesis involves a complex interplay of immune and inflammatory mechanisms, with pro-inflammatory cytokines such as tumor necrosis factor-alpha (TNF-α), interleukin-1 beta (IL-1β), and interleukin-6 (IL-6) playing central roles in driving disease progression and joint damage [4-7]. In addition, oxidative stress contributes significantly to tissue injury and amplifies inflammatory signaling, thereby accelerating disease severity.

Disease-modifying anti-rheumatic drugs (DMARDs), particularly methotrexate (MTX), remain the cornerstone of RA management. However, long-term therapy is often associated with adverse effects, including hepatotoxicity and gastrointestinal intolerance, which may limit patient adherence and therapeutic outcomes [8,9]. These limitations underscore the need for adjunctive strategies that can enhance efficacy while minimizing toxicity.

Psychological and physical stress are increasingly recognized as important modulators of RA severity [10-13]. Activation of the hypothalamic-pituitary-adrenal (HPA) axis and the sympathetic nervous system during stress leads to the release of glucocorticoids and catecholamines [14]. While acute stress responses may transiently regulate immune function, chronic stress can induce glucocorticoid resistance, impair endogenous anti-inflammatory mechanisms, and promote sustained cytokine production [15]. At the molecular level, stress-induced inflammation is closely associated with activation of nuclear factor-kappa B (NF-κB), a key transcription factor regulating inflammatory and oxidative pathways [16].

Eucalyptol (1,8-cineole), a monoterpene derived from Eucalyptus essential oils, has demonstrated anti-inflammatory, antioxidant, and analgesic properties [17]. It has been shown to impede pro-inflammatory cytokines via tempering of NF-κB signaling and clampdown of cyclooxygenase-2 (COX-2) and 5-lipoxygenase (5-LOX) pathways, while enhancing endogenous antioxidant defenses.

Despite growing evidence supporting the effect of stress in RA exacerbation and the beneficial effect of eucalyptol, their combined interaction remains insufficiently explored. Consequently, the present study aims to evaluate the protective effects of eucalyptol in a restraint stress-augmented Complete Freund’s Adjuvant (CFA)-induced arthritis model, with a focus on inflammatory, oxidative, molecular, and functional outcomes.

## Materials and methods

Experimental animals

Adult Wistar rats of mixed sex (8-10 weeks old, weighing 180-220 g), comprising equal numbers of males and females (50% each), were used in this study. Equal sex distribution was maintained across all experimental groups to minimize sex-related variability and reduce bias. Animals were housed in a controlled environment under a 12-hour light-dark cycle with access to food and water ad libitum. Rats were acclimatized for one week before experimentation.

All experimental procedures were approved by the Institutional Animal Ethics Committee (IAEC), KGMU, Lucknow (Approval No. 203/IAEC/Pharma/2024), and conducted in accordance with the guidelines of the Committee for the Control and Supervision of Experiments on Animals (CCSEA).

Drugs and chemicals

Eucalyptol and MTX were used as therapeutic agents. Arthritis was induced using Complete Freund’s Adjuvant (CFA) containing heat-killed Mycobacterium tuberculosis (Sigma-Aldrich, St. Louis, MO). Ethylenediaminetetraacetic acid (EDTA) was procured from Merck (Darmstadt, Germany). Serum levels of TNF-α, IL-4, IL-6, IL-10, NF-κB, and mitogen-activated protein kinase (MAPK) were quantified using commercially available enzyme-linked immunosorbent assay (ELISA) kits (Krishgen Biosystems, Mumbai, India). All other reagents were of analytical grade.

Experimental design

Rats were divided into eight groups (*n* = 6 per group) and subjected to a 28-day experimental protocol. Animals were assigned to different groups using the Microsoft Excel RAND function to ensure unbiased allocation. Group I served as the normal control and was given saline only. Group II consisted of CFA-induced arthritic rats without treatment. Group III included rats subjected to both CFA induction and restraint stress (RS) and served as disease control. Group IV received MTX (1 mg/kg) following CFA and RS exposure. Groups V, VI, and VII were treated with eucalyptol at doses of 50, 100, and 200 mg/kg (p.o.), respectively, in addition to CFA and RS. Group VIII received combination therapy consisting of eucalyptol (200 mg/kg) and MTX (1 mg/kg) under CFA and RS conditions. Drugs were administered intraperitoneally from Day 14 to Day 28.

Induction of arthritis and RS

Experimental arthritis was induced by intradermal injection of 0.2 mL CFA [18]. Prior sensitization was performed using 0.1 mL squalene [19]. Chronic stress was induced using an RS model, wherein animals were immobilized in well-ventilated cylindrical restrainers for three hours daily throughout the 28-day experimental period. The three-hour duration was selected based on published evidence that RS induces activation of the hypothalamic-pituitary-adrenal (HPA) axis, with peak corticosterone levels and downstream NF-κB-mediated neuroinflammatory signaling maximally sustained during this three-hour window [20-22].

At study termination (Day 28), animals were euthanized using pentobarbitone sodium (100 mg/kg, i.p.), and blood samples were collected and centrifuged at 3,000 rpm for 5 minutes for serum separation.

The doses of eucalyptol employed in this study were selected based on previously published dose-response studies demonstrating anti-inflammatory and antioxidant efficacy in rodent models [23]. MTX (1 mg/kg) was selected as the standard DMARD comparator based on its established clinical and experimental use in RA models. 

Assessment of physiological and behavioral parameters

Disease progression was evaluated on Days 0, 7, 14, 21, and 28 using clinical, physiological measurements, and behavioral assessments.

Arthritic index

Arthritis severity was scored using a standardized scoring system ranging from 0 to 16 based on erythema, edema, and joint deformity [24]. A score ≥1 in any non-injected paw was considered indicative of arthritis [25].

Paw volume and ankle diameter

Paw edema was measured using a digital plethysmometer, and ankle diameter was assessed using a digital vernier caliper [26].

Systemic parameters

Body weight and rectal temperature were recorded weekly.

Behavioral assessment

Locomotor activity was evaluated using the Any-maze video tracking system (Stoelting, USA), measuring total distance traveled and average speed. Neuromuscular strength was assessed using a grip strength meter, recording peak force (g).

Biochemical and molecular analysis

Oxidative Stress Markers

Lipid peroxidation (MDA) in paw tissue homogenates was estimated using the method of Ohkawa et al. [27]. Superoxide dismutase (SOD) activity was measured using the method of Marklund and Marklund [28]. Protein concentration was determined using Lowry’s method [29].

Nitrosative Stress and ROS

Total nitrite (NOx) levels were measured using the Griess reaction [30]. Reactive oxygen species (ROS) levels were quantified fluorometrically using the 2′,7′-dichlorodihydrofluorescein diacetate (DCFH-DA) probe [31].

Inflammatory and Anti-inflammatory Cytokines

Serum levels of TNF-α, IL-6, IL-4, and IL-10 were quantified using commercially available ELISA kits according to the manufacturer’s instructions.

*NF-κB​*​​​​​​* and MAPK Analysis*

Serum NF-κB and MAPK levels were measured using commercially available ELISA kits according to the manufacturer’s instructions and expressed in pg/mL.

qRT-PCR Analysis

Total RNA was isolated from joint tissues using TRIzol reagent, followed by reverse transcription to synthesize cDNA. Quantitative real-time polymerase chain reaction (qRT-PCR) was performed using SYBR Green chemistry. Expression levels of RT1A and matrix metalloproteinase-9 (MMP-9) were normalized to glyceraldehyde-3-phosphate dehydrogenase (GAPDH) and calculated using the comparative 2^−ΔΔCt method [32].

**Table 1:** Primer sequences used for quantitative real-time polymerase chain reaction (qRT-PCR).

Gene	Direction	Primer sequence (5'–3')
RT1A	Forward	GGC TGT GGA TGT TAC CCA GA
RT1A	Reverse	TCA GCA GGT GAC TTC CAA GA
MMP-9	Forward	TGG AAG CAA GAG TGG AGA GG
MMP-9	Reverse	GCA GCA GTA GGA GAT GGG AA

Histopathological studies

Ankle joints were collected on Day 28, fixed in 10% neutral-buffered formalin, decalcified in formic acid, and embedded in paraffin. Sections (5 µm) were stained with hematoxylin and eosin (H&E) and examined under a light microscope for synovial hyperplasia, inflammatory cell infiltration, pannus formation, cartilage damage, and bone erosion.

Statistical analysis

Data are expressed as mean ± standard deviation (SD). The Shapiro-Wilk test was used to assess normality, and Levene’s test was used to check for homogeneity of variance. One-way analysis of variance (ANOVA) followed by Tukey's post hoc test was used for comparisons between different groups at the same point in time, while two-way repeated-measures ANOVA with Bonferroni post hoc correction was used for repeated measures across time, to assess both the effects of treatment and of time on the outcome variable. Nonparametric tests (Kruskal-Wallis followed by Dunn's post hoc test) were used when the criteria for normality were not satisfied. GraphPad Prism (version 10; GraphPad Software, San Diego, CA) was used to perform all statistical analyses, and a *P*-value of <0.05 was considered statistically significant.

## Results

Effect of treatment on arthritic index and grip strength

All rats developed and progressed in terms of their arthritic index based on CFA induction over the course of 28 days, with RS serving to further exacerbate this development. The RS + CFA group showed statistically significant differences from the CFA-only group in terms of their arthritic index values. For example, Day 28 values for the arthritic index in the RS + CFA group were 15.9 ± 1.3, while the CFA-only group was 14.0 ± 1.0 (*P* < 0.05). There was also a significant reduction in grip strength (42 ± 4.6 g vs. 68 ± 4.3 g in CFA; *P* < 0.001), clearly indicating a functional impairment due to RA.

**Figure 1:**
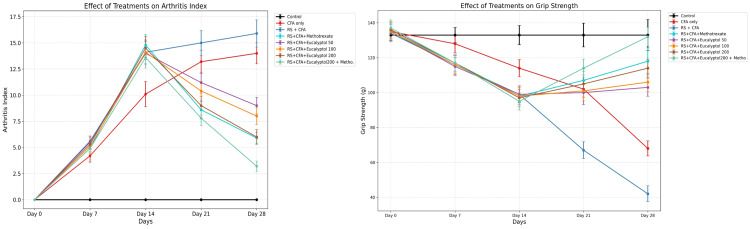
Effect of treatment on (A) arthritic index and (B) grip strength across experimental groups. Data are expressed as mean ± SD (*n* = 6). The arthritic index was analyzed using two-way repeated-measures ANOVA followed by Tukey’s post hoc test, while grip strength was analyzed using one-way ANOVA followed by Tukey’s post hoc test. Statistical significance: *P < 0.05 vs. CFA group; #*P* < 0.05 vs. RS + CFA group. CFA, Complete Freund’s Adjuvant; RS, Resveratrol; SD, standard deviation; n, sample size; ANOVA, analysis of variance; HSD, Honestly Significant Difference

Treatment with eucalyptol resulted in a dose-dependent reduction in disease severity. In the case of 200 mg/kg eucalyptol, the arthritic index reduced to 6.0 ± 0.7, while grip strength improved to 114 ± 5.2 g. The combination treatment of eucalyptol at 200 mg/kg and MTX yielded the most significant reduction in arthritic index (3.2 ± 0.5) as well as grip strength (132 ± 5.3 g vs. control: 133 ± 8.9 g) (Figures 1A-1B).

Effect of treatment on paw volume and ankle diameter

**Figure 2:**
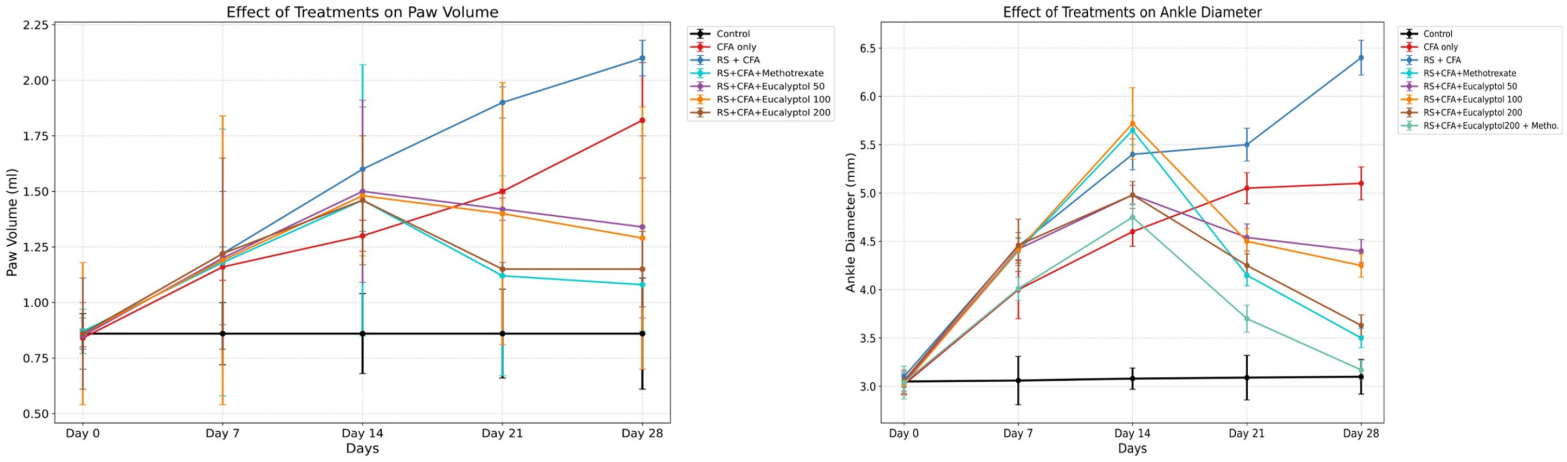
Effect of treatment on (A) paw volume and (B) ankle diameter. Data are presented as mean ± SD (n = 6). Paw volume and ankle diameter were measured over the experimental period. Statistical analysis was performed using two-way repeated-measures ANOVA followed by Tukey’s post hoc test. Statistical significance: *P < 0.05 vs. CFA group; #*P* < 0.05 vs. RS + CFA group. CFA, Complete Freund’s Adjuvant; RS, restraint stress; SD, standard deviation; n, sample size; ANOVA, analysis of variance; HSD, Honestly Significant Difference

Significant increases in paw volume and ankle diameter were observed in arthritic animals, with maximal severity in the RS + CFA group (2.10 ± 0.08 mL and 6.40 ± 0.18 mm, respectively). Eucalyptol treatment significantly reduced both parameters in a dose-dependent manner. At 200 mg/kg, paw volume and ankle diameter decreased to 1.15 ± 0.17 mL and 3.63 ± 0.11 mm, respectively, comparable to MTX treatment. Combination therapy further improved these outcomes, approaching normal control values (Figures 2A-2B).

Effect of treatment on locomotor activity

**Figure 3:**
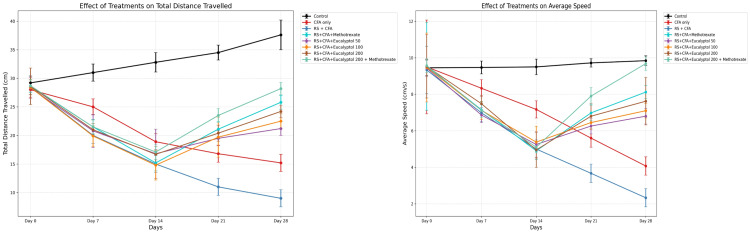
Effect of treatment on (A) total distance travelled and (B) average speed in the open-field test. Data are expressed as mean ± SD (*n* = 6). Statistical comparisons were performed using one-way ANOVA followed by Tukey’s post hoc test. Statistical significance: *P* < 0.05 vs. CFA group; #IP < 0.05 vs. RS + CFA group. CFA, Complete Freund’s Adjuvant; RS, restraint stress; SD, standard deviation; ANOVA, analysis of variance; HSD, Honestly Significant Difference

Locomotor activity was markedly impaired in the RS + CFA group, as indicated by reduced total distance traveled (9.0 ± 1.5 cm) and average speed (2.33 ± 0.50 cm/s). Eucalyptol treatment significantly improved locomotor performance, with the 200 mg/kg dose increasing distance traveled to 24.2 ± 1.1 cm and speed to 7.62 ± 1.3 cm/s. The combination group showed the greatest recovery (28.2 ± 1.1 cm and 9.68 ± 0.37 cm/s) (Figures 3A-3B).

Effect of treatment on body weight and body temperature

The RS + CFA group exhibited significant weight loss, with body weight declining to 152 ± 6.5 g by Day 28, compared to weight gain in controls (234 ± 7.1 g). Eucalyptol treatment mitigated weight loss, with the 200 mg/kg group reaching 197 ± 6.6 g and the combination group showing further improvement (204 ± 6.8 g) (Appendix A). A significant increase in body temperature was observed in the RS + CFA group (38.8 ± 0.14 °C), indicating systemic inflammation. Eucalyptol (200 mg/kg) reduced body temperature to 37.3 ± 0.09 °C, comparable to MTX-treated animals (body weight, Appendix A; body temperature, Appendix B).

Effect of treatment on oxidative stress markers

**Figure 4:**
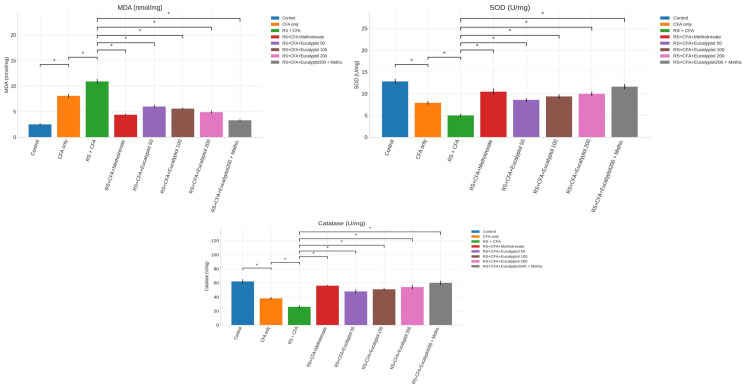
Effect of treatment on oxidative stress markers. Levels of malondialdehyde (MDA), superoxide dismutase (SOD), and catalase in paw tissue homogenates are presented as mean ± SD (n = 6). Statistical analysis was conducted using one-way ANOVA followed by Tukey’s post hoc test. Statistical significance: *P < 0.05 vs. CFA group; #*P* < 0.05 vs. RS + CFA group. CFA, Complete Freund’s Adjuvant; RS, restraint stress; MDA, malondialdehyde; SOD, superoxide dismutase; SD, standard deviation; ANOVA, analysis of variance; HSD, Honestly Significant Difference

The RS + CFA group showed marked oxidative stress, evidenced by elevated malondialdehyde (MDA) levels (10.9 ± 0.5 nmol/mg protein) and reduced antioxidant enzyme activities (SOD: 5.0 ± 0.4 U/mg protein; catalase: 26 ± 2 U/mg protein). Eucalyptol treatment significantly restored oxidative balance. At 200 mg/kg, MDA levels decreased to 4.9 ± 0.3 nmol/mg protein, while SOD and catalase activities increased to 10.0 ± 0.5 U/mg protein and 54 ± 3 U/mg protein, respectively (Figure 4).

Effect of treatment on nitrosative stress and ROS

**Figure 5:**
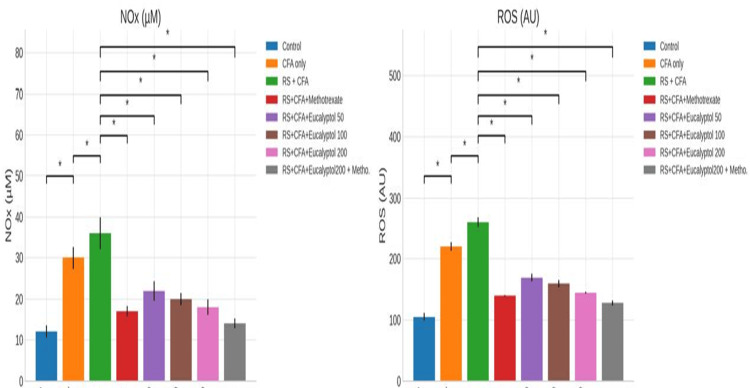
Effect of treatment on nitrosative stress and ROS. Levels of nitrite (NOx) and reactive oxygen species (ROS) in paw tissue homogenates are presented as mean ± SD (n = 6). Statistical analysis was performed using one-way ANOVA followed by Tukey’s post hoc test. Statistical significance: *P < 0.005 vs. CFA group; #*P* < 0.005 vs. RS + CFA group. CFA, Complete Freund’s Adjuvant; RS, restraint stress; SD, standard deviation; ANOVA, analysis of variance; HSD, Honestly Significant Difference

Nitrosative stress and ROS levels were significantly elevated in the RS + CFA group (NOx: 36 ± 3.9 µM; ROS: 260 ± 8.2 AU). Eucalyptol treatment significantly reduced these markers, with the 200 mg/kg dose lowering NOx to 18 ± 1.9 µM and ROS to 145 ± 1.5 AU. Combination therapy produced further reductions, approaching control levels (Figure 5).

Effect of treatment on pro-inflammatory and anti-inflammatory cytokines

**Figure 6:**
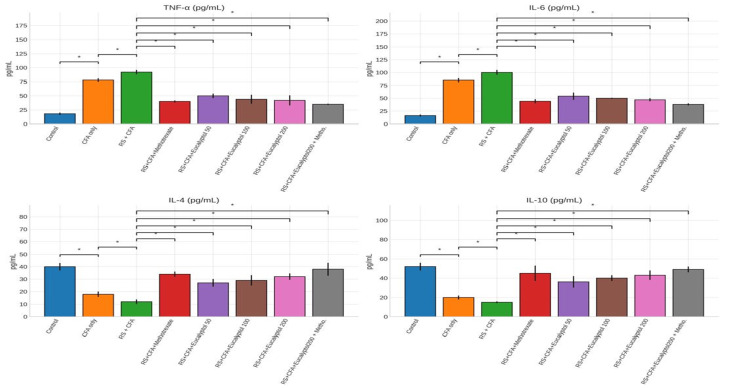
Effect of treatment on pro-inflammatory and anti-inflammatory cytokines. Serum levels of TNF-α, IL-6, IL-4, and IL-10 across experimental groups are expressed as mean ± SD (*n* = 6). Statistical comparisons were made using one-way ANOVA followed by Tukey’s post hoc test. Statistical significance: *P < 0.05 vs. CFA group; #*P* < 0.05 vs. RS + CFA group. CFA, Complete Freund’s Adjuvant; RS, restraint stress; TNF-α, tumor necrosis factor alpha; IL, interleukin; SD, standard deviation; ANOVA, analysis of variance; HSD, Honestly Significant Difference

The RS + CFA group exhibited significantly elevated TNF-α (92 ± 4 pg/mL) and IL-6 (100 ± 5 pg/mL), along with reduced IL-4 and IL-10 levels. Eucalyptol treatment significantly reduced pro-inflammatory cytokines and increased IL-10 levels (43 ± 5 pg/mL at 200 mg/kg). The combination therapy demonstrated the most pronounced immunomodulatory effect, restoring cytokine balance toward an anti-inflammatory profile (Figure 6).

Effect of treatment on serum NF‑κB and MAPK levels

**Figure 7:**
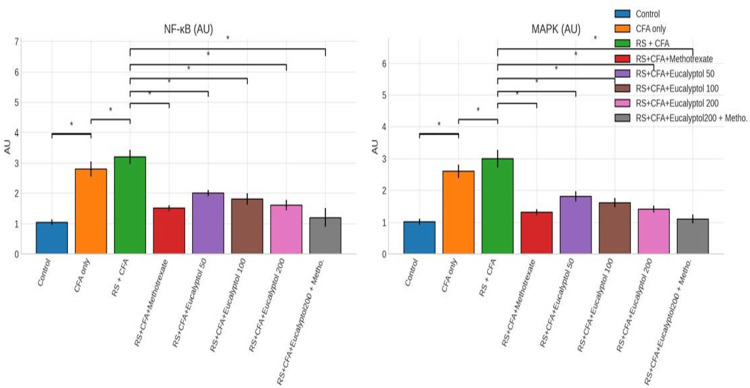
Effect of treatment on serum NF-κB and MAPK levels. Serum levels of NF-κB and MAPK signaling markers are expressed as mean ± SD (*n* = 6). Statistical analysis was performed using one-way ANOVA followed by Tukey’s post hoc test. Statistical significance: *P < 0.05 vs. CFA group; #*P* < 0.05 vs. RS + CFA group. CFA, Complete Freund’s Adjuvant; RS, restraint stress; NF-κB, nuclear factor kappa B; MAPK, mitogen-activated protein kinase; SD, standard deviation; ANOVA, analysis of variance; HSD, Honestly Significant Difference

Serum NF‑κB and MAPK levels were significantly elevated in the RS + CFA group (3.2 ± 0.24 AU and 3.0 ± 0.28 AU, respectively; *P* < 0.05). Eucalyptol treatment, alone and in combination with MTX, significantly suppressed activation of both signaling pathways, with maximal inhibition observed in the combination group (Figure 7).

Effect of treatment on gene expression

**Figure 8:**
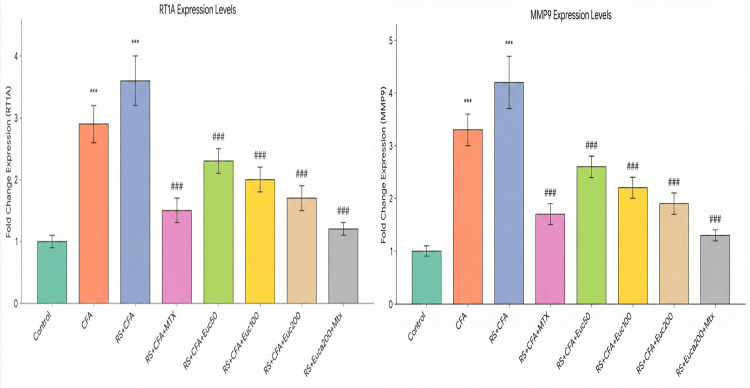
Effect of treatment on gene expression. Relative mRNA expression levels of RT1A and MMP-9 genes are expressed as mean ± SD (*n* = 6). Statistical comparisons were performed using one-way ANOVA followed by Tukey’s post hoc test. Statistical significance: **P < 0.001 vs. CFA group; ###P < 0.001 vs. RS + CFA group. CFA, Complete Freund’s Adjuvant; RS, restraint stress; mRNA, messenger ribonucleic acid; RT1A, rat major histocompatibility complex class I antigen; MMP-9, matrix metalloproteinase 9; SD, standard deviation; ANOVA, analysis of variance; HSD, Honestly Significant Difference

RT1A and MMP-9 mRNA expression levels were significantly increased in the RS + CFA group (3.6 ± 0.4-fold and 4.2 ± 0.5-fold, respectively) compared to controls. MTX reduced expression levels (RT1A: 1.9 ± 0.2-fold; MMP-9: 2.1 ± 0.3-fold), while eucalyptol treatment produced a dose-dependent decrease, with the highest dose (200 mg/kg) reducing RT1A and MMP-9 expression to 1.6 ± 0.2-fold and 1.8 ± 0.2-fold, respectively. The combination therapy showed the greatest effect, reducing expression levels to near-control values (RT1A: 1.2 ± 0.1-fold; MMP-9: 1.3 ± 0.1-fold) (Figure 8; Appendices C-D; Appendices E-F).

Effect of treatment on histopathological changes

Histopathological analysis revealed severe joint damage in CFA and RS + CFA groups, characterized by synovial hyperplasia, inflammatory infiltration, pannus formation, and cartilage erosion, with greater severity in the presence of stress.

**Figure 9:**
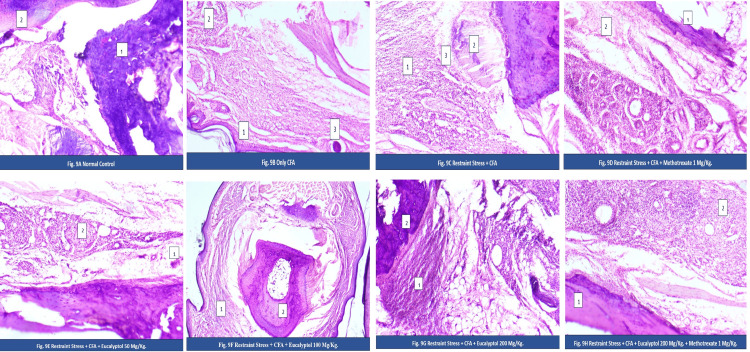
Effect of treatment on histopathological changes. Histopathological evaluation of ankle joint tissues across experimental groups: (A) Control rats showing normal synovial space, synovial lining, articular cartilage, and subchondral bone (no evident pathology). (B) Adjuvant group: (1) synovial hyperplasia; (2) prominent lymphocytic infiltration of the synovium with invasion of periarticular bone and vacuolization; (3) collapse of the articular surface and bone destruction. (C) Adjuvant + restraint stress: (1) inflammatory infiltrates; (2) expanding pannus; (3) collapse of the articular surface and bone destruction. (D) RS + CFA + MTX: (1) reduced cartilage damage; (2) decreased inflammatory infiltrates. (E) Eucalyptol (50 mg/kg): (1) giant cells; (2) reduced inflammation with fibrotic changes and negligible articular damage. (F) Eucalyptol (100 mg/kg): (1) minimal infiltration; (2) improved synovium. (G) Eucalyptol (200 mg/kg): (1) nearly normal synovium; (2) preserved cartilage. (H) Eucalyptol (200 mg/kg) + MTX: (1) normal cartilage; (2) minimal to no infiltration. CFA, Complete Freund’s Adjuvant; RS, restraint stress; MTX, methotrexate

Histopathological examination of the ankle joint tissue of the control group demonstrated the normal anatomically arranged joint consisting of an intact cartilage surface, normal synovial membrane, and subchondral bone, and the absence of any infiltrating immune cells (Figure 9A). The specimens from the CFA group were characterized by several pathologic features showing evidence of a severe disease state: (1) hyperplastic synovial membrane; (2) hemorrhage and extensive inflammatory cell infiltration; and (3) destruction of cartilage and the remaining adjacent subchondral bone (Figure 9B).

The degree of pathologic alteration in the RS + CFA group (Figure 9C) was considerably greater than that of the CFA group. There were also extensive infiltrating inflammatory cells, marked expansion of pannus tissue, and dilation of blood vessels in the joint tissue, as well as considerable loss of articular cartilage as a result of the additional physical stress factors associated with restraint (RS), which have caused an increase in the severity of the inflammatory arthritic condition. The treated animals receiving MTX (RS + CFA + MTX) (Figure 9D) showed marked decreases in all of the aforementioned pathologic alterations (i.e., within the RS + CFA group) and included decreased number of infiltrating inflammatory cells; partially restored normal synovial morphology; and reduced cartilage damage.

Eucalyptol administered at 50 mg/kg demonstrated a moderate protective effect (Figure 9E), with evidence of decreased synovial tissue thickening, reduced inflammatory cell proliferation, and minimal structural damage. The administration of eucalyptol at 100 mg/kg demonstrated enhanced protective effects as compared to the 50 mg/kg group and included additional reductions in inflammatory cell infiltrate and enhanced organization of synovial tissue (Figure 9F). The maximum therapeutic effect was observed in the eucalyptol 200 mg/kg group where the architecture of the joint was nearly restored (preservation of cartilage, marked decrease in inflammatory cell infiltrate, and normal morphology of synovial tissue, as depicted in Figure 9G). The most significant histological improvement was observed in the combination group eucalyptol 200 + MTX (Figure 9H), where joint architecture was largely restored, showing intact cartilage, minimal to absent inflammatory infiltration.

Histopathological evaluations showed that eucalyptol protected the joints from CFA- and stress-induced damage in a dose-dependent manner. Eucalyptol also produced statistically greater protection from CFA- and stress-induced joint damage than higher doses and had therapeutic effects comparable to traditional treatment with MTX.

## Discussion

The present study demonstrates that chronic restraint stress significantly exacerbates the clinical, biochemical, molecular, and histopathological manifestations of RA. At the same time, eucalyptol effectively mitigates these effects through coordinated anti-inflammatory and antioxidant mechanisms. Notably, the combination of eucalyptol with MTX produced superior therapeutic outcomes, suggesting a synergistic interaction.

RA is characterized by persistent synovial inflammation and progressive joint destruction driven by pro-inflammatory cytokines. In the current study, CFA-induced arthritis produced classical pathological features, including paw edema and joint rigidity, which were markedly intensified by restraint stress. The elevated arthritic index and ankle swelling observed in the RS + CFA group are consistent with previous reports indicating that stress-induced activation of the hypothalamic-pituitary-adrenal axis and sympathetic nervous system promotes immune dysregulation and enhances pro-inflammatory responses [33,34].

Functional impairment is a key feature of RA and reflects both inflammatory burden and joint damage. Rats exposed to combined stress and arthritis exhibited pronounced deficits in locomotor activity and grip strength, indicating severe functional limitation. Eucalyptol treatment significantly improved these parameters, suggesting both anti-inflammatory and analgesic effects. These improvements may be partially mediated through modulation of transient receptor potential (TRP) channels, particularly TRPM8, which has been implicated in monoterpene-mediated analgesia [35,36].

Oxidative stress plays a pivotal role in RA pathogenesis by amplifying inflammatory signaling and promoting tissue damage [37]. In this study, stress exposure markedly increased lipid peroxidation and reduced antioxidant enzyme activity, indicating severe oxidative imbalance. Eucalyptol effectively restored redox homeostasis by reducing malondialdehyde levels and enhancing superoxide dismutase and catalase activities. These findings are consistent with previous studies demonstrating the free radical-scavenging and cytoprotective properties of 1,8-cineole [38].

Chronic inflammation in RA is driven at the molecular level by the ongoing activation of NF-κB and MAPK signal transduction pathways, which modulate the cytokine gene expression profile of TNF-α and IL-6 [39]. Our results also support how stress exacerbates the production of these inflammatory markers, thereby increasing the degree of the inflammatory response. In addition, we demonstrated that treatment with eucalyptol significantly decreased serum levels of NF-κB and MAPK, suggesting that eucalyptol modulates these two signaling pathways. Although serum levels of NF-κB and MAPK provide evidence of potential signaling pathway involvement, additional direct and mechanistic-based studies that assess the inhibition of NF-κB and MAPK signaling pathways at the tissue level would provide additional support for these data [40].

Histopathological evaluations showed that eucalyptol protected the joints from CFA- and stress-induced damage in a dose-dependent manner. Both MTX alone and eucalyptol alone produced meaningful improvement in synovial inflammation and joint architecture compared to disease controls (RS + CFA group). Eucalyptol at the higher dose (200 mg/kg) demonstrated comparable or slightly greater preservation of joint tissue architecture than MTX alone; however, these differences did not reach statistical significance, indicating broadly similar histopathological efficacy between the two monotherapy groups. The combination of eucalyptol (200 mg/kg) and MTX produced the most pronounced protective effect, suggesting a potential additive or complementary interaction at the tissue level.

Gene expression analysis further supported these findings. Elevated RT1A and MMP-9 expression in the RS + CFA group reflects enhanced immune activation and extracellular matrix degradation. Both MTX and eucalyptol reduced gene expression levels, with eucalyptol demonstrating a dose-dependent effect. Importantly, combination therapy resulted in near-normalization of gene expression, suggesting a synergistic modulation of inflammation-associated molecular pathways.

MTX was chosen as the standard comparator in this study due to its well-established status as a first-line disease-modifying antirheumatic drug (DMARD) that inhibits underlying immune-inflammatory pathways (i.e., NF-κB and MAPK signaling pathways).In contrast, nonsteroidal anti-inflammatory drugs (NSAIDs) provide only symptomatic relief through COX inhibition. Therefore, eucalyptol's potential to modify the disease process of RA would be better compared to disease-modifying agents like MTX, rather than an NSAID (which is only used to relieve symptoms, but would not modify the disease). This is why MTX has been chosen as the appropriate comparator in this study to test eucalyptol for several reasons.

The combination of eucalyptol and MTX consistently produced superior outcomes across clinical, functional, biochemical, and histological parameters. This suggests a complementary mechanism in which eucalyptol’s antioxidant and immunomodulatory properties enhance the therapeutic efficacy of MTX. Such combination strategies may allow optimization of DMARD therapy, potentially reducing long-term toxicity and improving patient outcomes [41,42].

Histopathological findings further corroborated these results. Stress markedly intensified synovial hyperplasia, pannus formation, and cartilage erosion, supporting previous evidence linking neuroendocrine stress to accelerated joint destruction [43]. While MTX partially attenuated these changes, residual structural damage persisted, highlighting the limitations of monotherapy [44,45]. In contrast, eucalyptol demonstrated dose-dependent chondroprotective effects, with the highest dose preserving joint architecture. The combination regimen achieved near-complete structural restoration, consistent with its inhibitory effects on inflammatory signaling and oxidative stress [46].

## Conclusions

In conclusion, this study shows that chronic restraint stress is a significant factor in exacerbating inflammatory arthritis and highlights the importance of neuroendocrine and immune interactions in the progression of the disease. Eucalyptol has an effect by reducing stress-induced RA through antioxidant and anti-inflammatory actions, and also showed evidence of its potential to be used as an adjunct to methotrexate - a first-line disease-modifying anti-rheumatic drug to target disease development - in the management of RA. Future studies are needed to further support these findings through sex-based statistical analysis and more directly demonstrating the modulation of relevant pathways to support their potential for clinical utility.

## Figures and Tables

**Figure 10:**
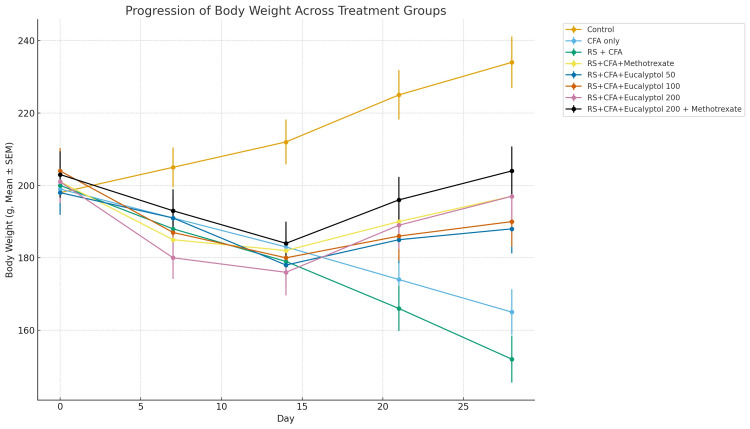
Effect of Treatment on Body Weight (Supplementary Figure 1 )

## Appendices

Appendix A 

Appendix B 

**Table 2:** (Table S1): Effect of treatment on Body Temperature (°C) Changes in body temperature (°C) across experimental groups. Data are expressed as mean ± standard deviation (SD) (n = 6 per group). Days 0–14 represent pre-treatment measurements, while Days 21–28 represent post-treatment measurements. *p < 0.05 versus the control group #p < 0.05 versus the CFA-only group.

Groups	Day 0	Day 7	Day 14	Day 21	Day 28
Control	37.1 ± 0.01	37.1 ± 0.03	37.2 ± 0.01	37.2 ± 0.02	37.1 ± 0.01
CFA only	37.1 ± 0.09	37.3 ± 0.10	37.7 ± 0.12^*^	38.0 ± 0.13^*^	38.3 ± 0.10^*^
RS + CFA	37.2 ± 0.09	37.9 ± 0.11^*^	38.2 ± 0.13^*^	38.5 ± 0.14^*^	38.8 ± 0.13^*^
RS + CFA + MTX (1 mg/kg)	37.2 ± 0.08	37.8 ± 0.20^*^	38.1 ± 0.11^*^	37.4 ± 0.11^#^	37.2 ± 0.05^#^
RS + CFA + Eucalyptol (50 mg/kg)	37.1 ± 0.09	37.9 ± 0.18^*^	38.2 ± 0.18^*^	37.7 ± 0.11^#^	37.5 ± 0.09^#^
RS + CFA + Eucalyptol (100 mg/kg)	37.2 ± 0.08	37.8 ± 0.45^*^	38.3 ± 0.11^*^	37.6 ± 0.10^#^	37.4 ± 0.09^#^
RS + CFA + Eucalyptol (200 mg/kg)	37.2 ± 0.08	37.9 ± 0.16^*^	38.1 ± 0.27^*^	37.5 ± 0.09^#^	37.3 ± 0.06^#^
RS + CFA + Eucalyptol (200 mg/kg) + MTX (1 mg/kg)	37.3 ± 0.08	37.8 ± 0.09^*^	38.2 ± 0.10^*^	37.3 ± 0.09^#^	37.2 ± 0.04^#^

Appendix C 

**Table 3:** (Table S 2a): Effect of treatment on RT1A mRNA Expression (Immune activation marker) Δ Ct Values RT1A mRNA expression (ΔCt values). Data are expressed as mean ± standard deviation (SD) (n = 6 per group). ΔCt values were calculated as the difference between target gene (RT1A) and the reference gene (GAPDH). Lower ΔCt values indicate higher gene expression.

Groups	Δ Ct (RT1A − GAPDH)
Control	7.90 ± 0.18
CFA	4.30 ± 0.16
RS + CFA	3.80 ± 0.15
RS + CFA + MTX	6.60 ± 0.16
RS + CFA + Euc. 50	5.40 ± 0.19
RS + CFA + Euc. 100	6.00 ± 0.18
RS + CFA + Euc. 200	6.50 ± 0.17
RS + CFA + Euc. 200 + MTX	7.10 ± 0.15

Appendix D 

**Table 4:** (Table S 2b): Effect of treatment on ΔΔ Ct and Fold Change (2⁻ΔΔCt) RT1A relative expression (ΔΔCt and fold change). Relative gene expression was calculated using the 2⁻ΔΔCt method, with the control group used as the calibrator. Fold change values represent relative expression normalized to GAPDH and expressed relative to control.

Groups	ΔΔ Ct	Fold Change
Control	0.00	1.00
CFA	−3.60	12.1 ↑
RS + CFA	−4.10	17.1 ↑↑
RS + CFA + MTX	−1.30	2.46 ↓
RS + CFA + Euc 50	−2.50	5.66 ↓
RS + CFA + Euc 100	−1.90	3.73 ↓
RS + CFA + Euc 200	−1.40	2.64 ↓
RS + CFA + Euc 200 + MTX	−0.80	1.74 ↓↓

Appendix E 

**Table 5:** (Table S 3a): Effect of treatment on MMP-9 mRNA Expression (Joint destruction marker)Δ Ct Values (Mean ± SEM) MMP-9 mRNA expression (ΔCt values). Data are expressed as mean ± standard deviation (SD) (n = 6 per group). ΔCt values were calculated as the difference between target gene (MMP-9) and GAPDH. Lower ΔCt values correspond to higher gene expression levels.

Groups	Δ Ct (MMP-9 − GAPDH)
Control	8.30 ± 0.20
CFA	3.90 ± 0.17
RS + CFA	3.30 ± 0.16
RS + CFA + MTX	6.50 ± 0.16
RS + CFA + Euc 50	5.00 ± 0.19
RS + CFA + Euc 100	5.80 ± 0.17
RS + CFA + Euc 200	6.20 ± 0.18
RS + CFA + Euc 200 + MTX	7.10 ± 0.15

Appendix F 

**Table 6:** (Table S 3b):Effect of treatment on MMP-9 relative expression (ΔΔCt and fold change). MMP-9 relative expression (ΔΔCt and fold change). Relative expression levels were determined using the 2⁻ΔΔCt method with the control group as the reference. Fold change values indicate relative gene expression normalized to GAPDH

Groups	ΔΔ Ct	Fold Change
Control	0.00	1.00
CFA	−4.40	21.1 ↑
RS + CFA	−5.00	32.0 ↑↑
RS + CFA + MTX	−1.80	3.48 ↓
RS + CFA + Euc 50	−3.30	9.85 ↓
RS + CFA + Euc 100	−2.50	5.66 ↓
RS + CFA + Euc 200	−2.10	4.29 ↓
RS + CFA + Euc 200 + MTX	−1.20	2.30 ↓↓
